# miR-451 Up-regulation, Induce Erythroid Differentiation of CD133+cells Independent of Cytokine Cocktails

**Published:** 2013-06

**Authors:** Fatemeh Kouhkan, Masoud Soleimani, Morteza Daliri, Mehrdad Behmanesh, Naser Mobarra, Majid Mossahebi mohammadi, Shahin Mohammad, Mehdi Mokhtari, Reyhaneh Lahmy

**Affiliations:** 1 *Department of Genetics, Faculty of Biological Sciences, Tarbiat Modares University, Tehran, Iran*; 2 *Department of Hematology, School of Medical Sciences, Tarbiat Modares University, Tehran, Iran*; 3 *National institute of genetic engineering and biotechnology, Tehran, Iran*; 4 *Department of Clinical biochemistry, School of Medicine, Tehran University of Medical Sciences, Tehran, Iran*; 5 *Department of Hematology, Allied Medical School, Tehran University of Medical Sciences, Tehran, Iran*; 6 *Departments of Epidemiology and Biostatistics, School of Public Health, Tehran University of Medical Sciences, Tehran, Iran*

**Keywords:** Erythropoiesis, CD133+, microRNA, miR-451, miR-16

## Abstract

***Objective(s):*** Erythropoiesis is regulated by some extrinsic and intrinsic factors as microRNAs (miRNAs). miRNAs are endogenously small non-coding regulatory RNAs which play vital roles in the variety of cellular fate, critical processes; growth, apoptosis, metabolism, survival of the cells and specially differentiation. Several miRNAs such as miR-16 and miR-451 have been shown to be correlated with erythroid differentiation. Taking into account the importance of miRNAs in cellular differentiation, the goal of the present study was to examine the role of miRNAs in hematopoietic stem cells (HSC) differentiation into the erythroid cells in the absence of growth factors and stimulatory cytokines.

***Materials and Methods:*** CD133+ stem cells were infected with lentiviruses containing miR-451/miR-16 precursor sequence, erythroid differentiation was evaluated using RT-PCR for hemoglobin chains and surface antigens, also by banzidine staining.

***Results:*** MiR-451up-regulation, but not miR-16, could induce α, β and γ-globin expression in CD133+ cells and have strong correlation with appearance of CD71 and CD235a markers in these cells. Moreover, miR-451 up-regulation increases the banzidine positive cells to ~ %40.

***Conclusion:*** Our results provide strong evidence that miR-451 up-regulation strongly induces erythroid differentiation and maturation of CD133+ stem cells. Hence, this method may provide a useful technique for the production of artificial blood RBC and be used as a new strategy for gene therapy of hemoglobinopathies, such as β-thalassemias and sickle cell anemia.

## Materials and Methods

***Recombinant lentiviruses production***

The pCDH-451 and pCDH-16 plasmids were generated by ligation of 250 and 458 bp fragments encompassing pri-miR-451 and pri-miR-16 sequences, respectively, into the XbaI /BamHI restriction sites of pCDH-CMV-MCS-EF1-copGFP vector (System Biosciences, USA). These fragments were amplified by PCR reaction using following primers: pri-miR-451 FW: 5′- GGAAGATCTTGACAAGGAGGACAGGAGAG -3′, pri-miR-451 RW: 5′- CCCAAGCTTGCCTTGTTTGAGCTGGAGTC-3′; pri-miR-16 FW: 5′- ACTCTAGAGCAGCACATAATGGTTTG-3′ and pri-miR-16 RW: 5′-TGGATCCCTCTAATGCTGCATAAGC- 3′ on genomic DNA extracted from human whole blood (Cinnagen, Iran). lentivirus production; human embryonic kidney (HEK) 293 cells (3 ×10^6^) were seeded into 10-cm plates containing DMEM medium supplemented with 10% FBS(purchased from Gibco). The next day, pPAX2 plasmid (containing *gag* and *pol *genes) and pMD2 plasmid (containing *vsv* gene) were co-transfected with pCDH-451/ pCDH-16 plasmids also empty vector (pCDH empty backbone) as negative control into seeded 293T cells using lipofectamin 2000 reagent (Invitrogen, USA) according to manufacturer’s protocol. The supernatants containing produced lentiviruses were collected every 12 hours for 2 days after transfection and concentrated by ultracentrifuge at 40.000g for 2 hr. Then for virus titration, 293T cells were transduced with a different concentration of recombinant lentiviruses and the number of viruses in the functional copy was determined using GFP protein and fluorescent microscope forty-eight hours later. 

***CD133+ cells isolation, culture and infection***

Human umbilical cord blood (UCB) was obtained from 3 healthy full-term placentas. Written permission was obtained from healthy pregnant women. Next, to purify CD133+ stem cells, MNCs (mononuclear cells) were isolated from UCB samples by density gradient centrifugation using Ficoll-paque reagent (GE Healthcare, Sweden). CD133+ cells were enriched through positive selection using CD133 magnetic micro-beads and MidiMACS separation systems (Miltenyi Biotec, Germany) as per manufacturer's recommendations. Enriched CD133+ stem cells were expanded into StemSpan media (Stem Cell Technologies, Canada) in the presence of 100ng/mL stem cell factor (SCF), 100 ng/mL thrombopoietin (TPO) and 100ng/mL Flt3-ligand (Stem Cell Technologies) for 5 days. For gain of function studies, expanded CD133^+^ cells were transferred at density of 1×10^5^ cells per well in 24-well plates containing IMDM media (purchased from Gibco) supplemented with only 5% FBS in the absence of any erythroid stimulatory growth factors and cytokines. The next day, CD133+ cells were infected with a multiplicity of infections (MOI of 30, It means that 30 lentivirus particles were added per one CD133+ stem cell). 

The study was performed in four groups: one group was transduced with pCDH-451 lentiviruses (pCDH-451 group), another was infected with pCDH-16 lentiviruses (pCDH-16 group), the third did not receive any treatment (blank control group) and the fourth group was transduced with pCDH-empty lentiviruses (negative control group). After 14 days, the role of miR-16 and miR-451 up-regulation in erythroid differentiation was monitored by hemoglobin chains (α, β, γ and ζ) RT-PCR, erythroid cell surface markers (CD71 and CD235a) RT-PCR and banzidine staining.

***RNA extraction and RT-PCR***

Total RNA was extracted from test and control groups using Biozol reagent (Bioer,China ) on day 14 post infection. The first strand cDNA was synthesized with AMV reverse transcriptase enzyme from 100 ng of total RNA using cDNA synthesis kit (Bioer) according to manufacturer’s instructions. Next, RT-PCR reactions were performed for hemoglobin genes (α, β, γ and ζ) and erythroid specific cell surface markers (transferrin receptor, CD71:120 bp and glycophorinA, CD235a:167 bp) as erythroid differentiation indexes using following primers: α-globin (annealing temperature: 59ْC, Product size:407 bp): FW, 5′- CCGACAAGACCAACGTCAAGG-3′ and RW, 5′-GGTATTTGGAGGTCAGCACG-3′; β-globin (annealing temperature: 60ْC, Product size:122 bp): FW, FW, 5′-CTCACCTGGACAACCTCAAG -3′; and RW, 5′-TACCCGGTCGTGTGTCTGGT-3′; γ-globin (annealing temperature: 54ْC, Product size: 194 bp): FW, GTCCTCTGC-CTCTGCCATC -3′ and RW, 5′-CGGTCACCAGCACATTTCC-3′; ζ-globin (annealing temperature: 55ْC, Product size: 208 bp): FW, 5′- GGTGAAGAGCATCGACGACA-3′ and RW, 5′-TCTCGGTCAGGACAGAGGA-3′; CD71 (annealing temperature: 60ْC, Product size: 120 bp): FW, 5′-TGAGGTGGCAATGCACACTTC-3′ and RW, 5′- AGGAGTGGCTGCATATGTGTCC-3′; CD235a (annealing temperature: 60ْC , Product size: 167 bp): FW, 5′- GGCTAAGGTCAGACACTGAC-3′ and RW, 5′- TGTGCATTGCCACCTCAGTG-3′; GAPDH (annealing temperature: 58ْC, Product size: 166 bp): FW, 5′- GACAAGCTTCCCGTTCTCAG -3′ and RW, 5′- GAGTCAACGGATTTGGTCGT.

***miRNA-qRT- PCR***

Total RNA was extracted from all samples at day 4. Then, qRT-PCR for miR-16 and miR-451 were performed using Stratagene kit (USA) according to manufacturer’s protocol. Briefly, first cDNA strand was synthesized through miRNA 1st-strand cDNA synthesis kit (Stratagene) and reverse transcribed into qPCR-ready cDNA. After that, miRNA qRT-PCR analysis was carried out in triplicate on ABI PRISM 7500 real time PCR System (Applied Biosystems, USA) with the high-specifity miRNA qPCR core reagent kit (Stratagene) using following miRNA-specific forward primers: miR-451 FW, 5-′ AAACCGTTACCATTACTGAGATT-3′ and miR-16 FW: 5′- AGCAGCACGTAAATATTGGC-3′ according to manufacturer’s instructions and normalized to U6 small nuclear RNA (snRNA) as endogenous control. U6 primers were: FW: 5′: CTCGCTTCGGCAGCACACATATAC-3′, RW: 5′- ACGCTTCACGAATTTGCGTGTC-3′. The qRT-PCR cycling conditions were 10 minutes at 95°C followed by 10 sec at 95°C, 15 sec at 60°C, and 20 sec at 72°C, repeated for 30 step cycles. Data analyses were performed using the relative quantification of CT method. 

***Banzidine staining***

Banzidine staining was used for calculation of percentage of cells which express hemoglobin chains. For producing benzidine solution, 5 mL of 30% hydrogen peroxide was added to 1 mL of a stock solution of 0.2% benzidine dissolved in 0.5% acetic acid. A number of 10^5^ cells from each test and control groups were resuspended in phosphate buffered saline (PBS) and mixed with the same volume of the benzidine solution for 7 min in room temperature. Then, each sample was monitored by light microscopy and the blue cells (benzidine-positive cells) were counted and expressed as a percentage. 100 cells were counted in each sample and all experiments were performed triplicate. 

***Statistical analysis***

All experiments were repeated three times and data were presented as mean±SD. The comparison between groups was performed by Student's t- test.* P- *value less than 0.05 designated statistically significant differences. 

## Results

***Erythroid differentiation of CD133+ cells***

**Figure 1:**
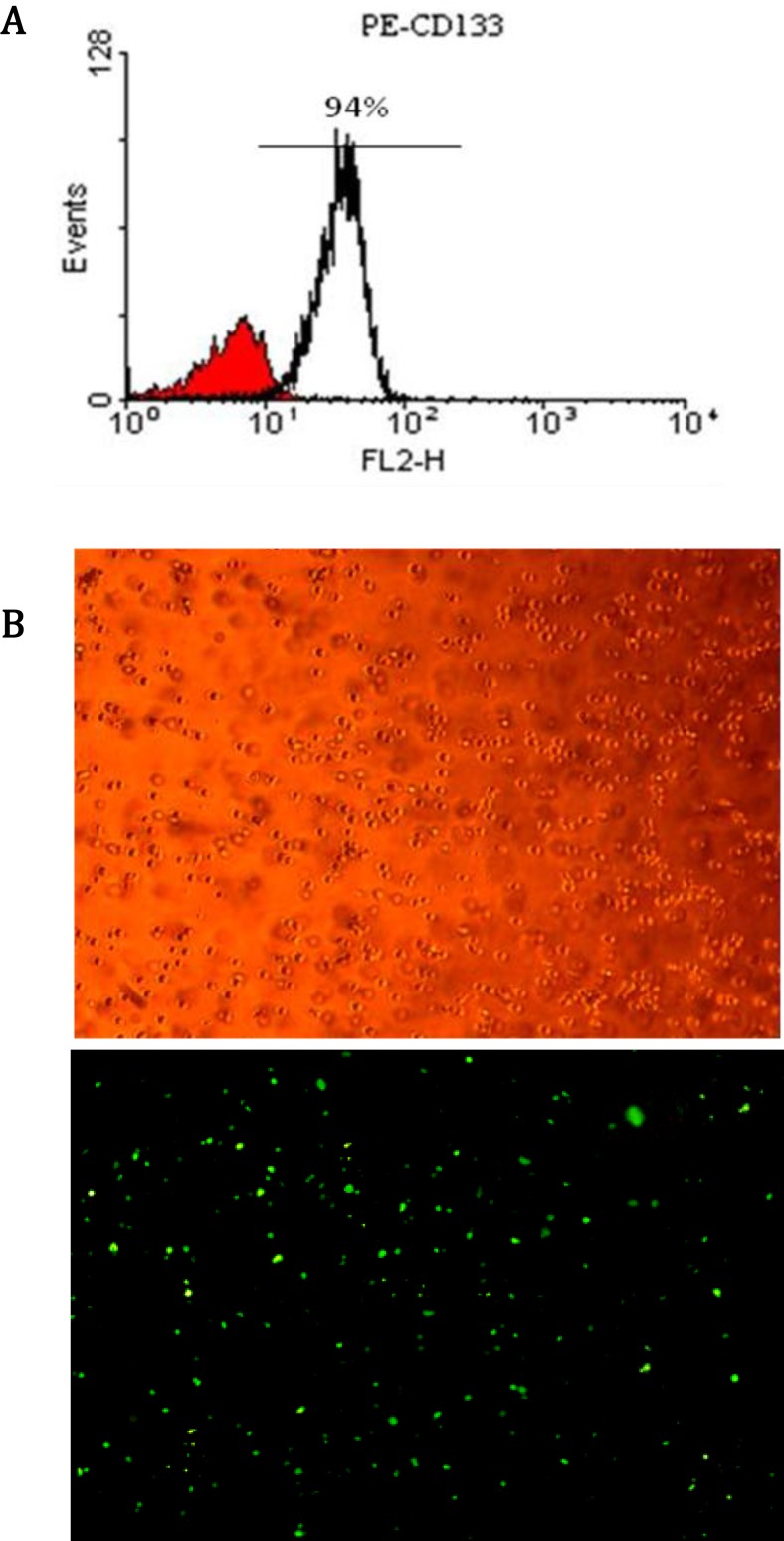
CD133+ stem cell purification and infection. A: Result of CD133+ cells purification from HCB by flow cytometry. In each case, purification efficiency was above 90%. B: (B, above) Transduced CD133+ cells examined by light microscopy. (B, below Transduced CD133+ cells examined by fluorescent microscopy. Transduction efficiency of CD133+ cells with pCDH-16 and pCDH-451 was obtained approximately more than 70% as determined by fluorescent microscopy

CD133+ stem cells were isolated successfully from each cord blood sample. The purity rate of isolated cells was measured by flow cytometry (Figure 1, A). Quantities over than 90% were selected for further studies. Then, CD133+ stem cells were successfully transduced by lentiviral vectors. Transduction efficiency was checked each time by fluorescent microscopy and determined about 70% (Figure 1, B).

***Recombinant lentiviruses increased mature miRNAs level in treated CD133+ cells***

To determine the efficacy of recombinant lentiviruses, we measured the expression level of miR-16 and miR-451 at day 4 after transduction in test and control groups by qRT-PCR. Treatment of CD133+ cells with pCDH-16 lentiviruses, but not with pCDH-empty lentiviruses, led to rise of mature miR-16 by 13-fold relative to the blank control cells 4 days upon transfection. Similar results were obtained when CD133+ cells were infected with pCDH-451 lentiviruses. Over expression of miR-451 in pCDH-451 group enhanced mature miR-451 by 11.5-fold compared with blank control group. As expected, when CD133+ cells were treated with pCDH-empty lentiviruses, miR-451 expression level showed no significant alteration compared to blank control group (*P*> 0.05, Figure 2). These results indicate that both recombinant lentiviruses are functional and increase mature miRNAs level appreciably.

***pCDH-451 lentiviruses, but not pCDH-16 lentiviruses, could induce erythroid differention of CD133+ cells***

Because miR-16 and miR-451were up-regulated drastically during erythroid differentiation, we sought to determine whether these miRNAs could induce erythropoiesis individually and in the absence of any erythroid stimulatory growth factors and cytokines. So, we evaluated the effect of mentioned miRNAs upregulation on expression of erythroid specific markers. CD133+ cells were infected with pCDH-451, pCDH-16 or pCDH-empty lentiviruses and erythroid differentiation was assessed by RT-PCR for hemoglobin chains and cell surface markers (Figure 2A and B). Over expression of the miR-451 induced expression of α, β and γ- globin, but not the expression of the ζ- globin. 

Noteworthy, γ- globin was expressed at lesser extent than α and β chains. However, miR-16 up-regulation in pCDH-16 group had no effect on the hemoglobin chains expression. Similarly, miR-451 up-regulation led to stimulation of CD71 and CD235a expression. Nevertheless, miR-16 up-regulation did not positively effect the expression of either cell surface marker. Since, β-globin and CD235a genes are the important markers of maturing human erythroid cells, these findings suggest that miR-451 up-regulation, but not miR-16 up-regulation, is able to promote erythroid differentiation of the HSCs and even its maturation without adding any stimulatory factors. 

**BFigure 2:**
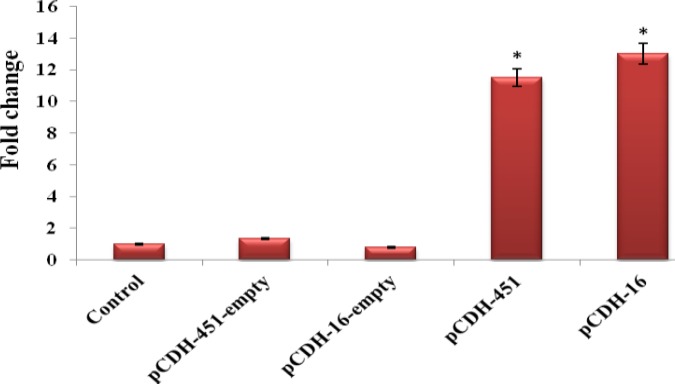
Expression of miR-16 and miR-451 in test and control groups was measured by QRT-PCR at day 4. The expression level of both miRNAs was assessed in the group that infected with pCDH-empty group. All tests were performed in triplicate and data were presented as mean ± SD. Error bars designate SD. * *P*<0.05

***miR-451 up-regulation correlated with banzidine positive cells***

**Figure 3:**
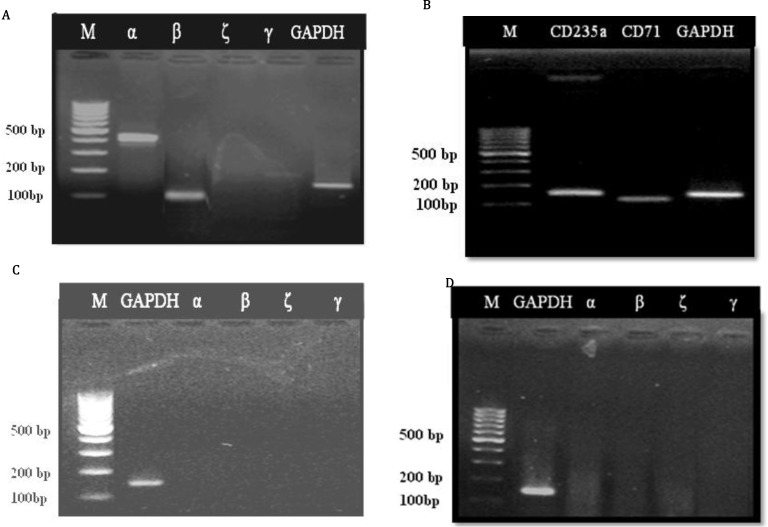
RT-PCR results of hemoglobin chains and surface CD markers expression in pCDH-451 group (A and B), control group (C) and pCDH-16 group (D). α-, β-, γ-globin, CD71 and CD235a were expressed in pCDH-451 group, but not in pCDH-16 group and control group. Product size of α: 407 bp, β: 122 bp, γ: 194 bp, ζ: 208 bp, GAPDH: 166 bp, CD71: 120 bp, CD235a: 167 bp, Marker (M): 100 bp

Erythroid differentiation and hemoglobinization were also examined by banzidine staining (Figure 3). MiR-451 over expression significantly increased the proportion of the cells which were benzidine-positive in pCDH-451 group versus control group at day 14 (40.2% vs. 4.7%, respectively), whereas miR-16 up-regulation had no obvious effect on the hemoglobinization (only 5.5% cells was benzidine-positive). Unsurprisingly, no significant alteration was seen between the percentage of the cells that were positive for banzidine staining in pCDH-empty group and blank control group (3.8% vs. 4.7%, respectively, *P*> 0.05). Taken together, these results further confirmed that miR-451 up-regulation contribute to erythroid differentiation of CD133+ cells and identify this miRNA as one of the main factors that induces hemoglobinization.

## Discussion

MicroRNAs (miRNAs) are small, non–protein-coding RNAs that recognize target sites, in the 3′ untranslated regions (UTRs) of cognate mRNAs, through imperfect base-pairing, and either destabilize them or inhibit protein translation. miRNAs is involved in different processes in multi-cellular plant and animal species but not in unicellular organisms including cell development, fat storage, apoptosis and differentiation (29-34). Essential roles of miRNAs also were identified in hematopoietic lineage differentiation (23-25). For instance, Bruchova *et al* (2007) performed gene-expressing profiling using microarray method and indicated that miR-16 and miR-451 up-regulated by 35-fold and 3-fold, respectively, throughout the erythroid differentiation (35-37). 

**Figure 4:**
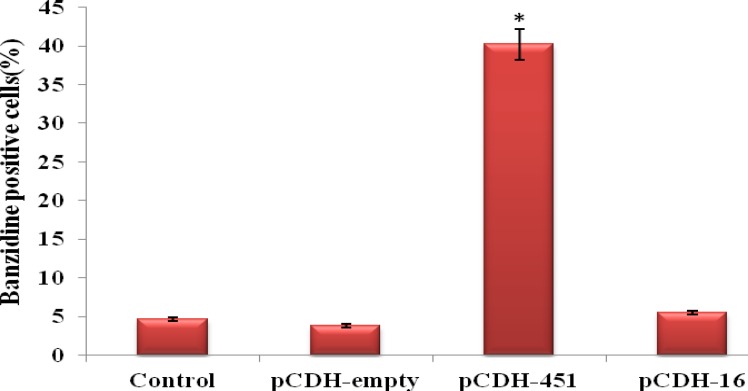
Evaluation of hemoglobinization using banzidine staining method. Infection of CD133+ cells with miR-451 increased banzidine positive cells to ~ 40%. In other group no obvious changes were observed. Results indicate the mean ± standard deviation of three independent experiments. Error bars represent SD. * *P*<0.05

In the present study, to determine whether miR-16 and miR-451 could play central roles in erythroid differentiation in the absence of cytokines and growth factors combination, we analyzed the effect of enforced expression of these miRNAs on erythroid differentiation of CD133+ cells. RT-PCR results indicated that, when cells were treated with pCDH-451 lentiviruses, miR-451 up-regulation persuaded the erythroid surface markers CD71 and CD235a. In addition miR-451 overexpression induced hemoglobin α and β expression at high level, whereas miR-451 stimulated γ-globin expression at low level. Findings demonstrate that in this model, adult β-globin (Hb A), with negligible fetal globin (Hb F) was produced that mimics *in vivo* erythropoiesis. Our results are consistent with a report that states up-regulation of miR-451, increased β-globin and hemoglobinization in MEL cells during DMSO-induced erythroid differentiation (19). Moreover, our data showed that the up-regulation of miR-451caused a significant rise in CD133+ hemoglobinization (~ %40). These findings suggest that miR-451 up-regulation alone and without adding any auxiliary factors could induce erythroid differentiation of CD133+ cells. However, miR-451 target(s) are not yet identified in humans (25). 

On the other hand, transduction of the cells with pCDH-16 lentiviruses could not induce hemoglobin and surface antigen genes expression in CD133+ cells. Our observations seem to be in disagreement with a previous study by Choong *et al* who demonstrated that miR-16 have strong positive correlation to the appearance of erythroid specific cell surface markers such as CD36, CD71 and CD235a and hemoglobin synthesis upon erythroid differentiation of CD34+ cells using cytokines combinations (27). These dissimilarities may be explained by differences in the method of erythropoietic induction of the stem cells and the source of progenitor cells. Thus, up-regulation of miR-16 on their own and without stimulatory cytokines assistance did not promote erythroid differentiation of the CD133+ cells.

## Conclusion

Taken together, our evidence suggests that miR-451 up-regulation maybe a critical factor for *in vitro *erythropoiesis of HSCs and production of artificial blood. On the other hand, in the hemoglobinopathies such as sickle cell anemia and thalassemia, the major problem is failure in the production of adult globin (HbA) (38). Thus, erythropoiesis using miR-451 and other miRNAs may be useful in studying the pathophysiology of hemoglobinopathies and test effective therapeutic strategies for the possibility of reversing these abnormalities by gene therapy. However, it is clear that for clinical applications, complete process of *in vitro* erythropoiesis using miRNAs expression modulation should be proved functionally and morphologically. Hence, present study is the primary step in this field and further studies would be necessary.
